# Comparative analysis of hypertensive nephrosclerosis in animal models of hypertension and its relevance to human pathology. Glomerulopathy

**DOI:** 10.1371/journal.pone.0264136

**Published:** 2022-02-17

**Authors:** Alex A. Gutsol, Paula Blanco, Taben M. Hale, Jean-Francois Thibodeau, Chet E. Holterman, Rania Nasrallah, Jose W. N. Correa, Sergey A. Afanasiev, Rhian M. Touyz, Chris R. J. Kennedy, Dylan Burger, Richard L. Hébert, Kevin D. Burns

**Affiliations:** 1 Ottawa Hospital Research Institute & Kidney Research Centre, University of Ottawa, Ottawa, ON, Canada; 2 Department of Pathology and Laboratory Medicine, University of Ottawa, Ottawa, ON, Canada; 3 Basic Medical Sciences Faculty, University of Arizona, Tucson, AZ, United States of America; 4 Department of Physiological Sciences, Biological Sciences Institute, Federal University of Amazonas, Manaus, Brazil; 5 Cardiology Research Institute, Tomsk, Russian Federation; 6 Research Institute of the McGill University Health Centre, McGill University, Montreal, QC, Canada; 7 Department of Cellular and Molecular Medicine, University of Ottawa, Ottawa, ON, Canada; 8 Division of Nephrology, Department of Medicine, University of Ottawa, Ottawa, ON, Canada; University Medical Center Utrecht, NETHERLANDS

## Abstract

Current research on hypertension utilizes more than fifty animal models that rely mainly on stable increases in systolic blood pressure. In experimental hypertension, grading or scoring of glomerulopathy in the majority of studies is based on a wide range of opinion-based histological changes that do not necessarily comply with lesional descriptors for glomerular injury that are well-established in clinical pathology. Here, we provide a critical appraisal of experimental hypertensive glomerulopathy with the same approach used to assess hypertensive glomerulopathy in humans. Four hypertensive models with varying pathogenesis were analyzed–chronic angiotensin II infused mice, mice expressing active human renin in the liver (TTRhRen), spontaneously hypertensive rats (SHR), and Goldblatt two-kidney one-clip rats (2K1C). Analysis of glomerulopathy utilized the same criteria applied in humans–hyalinosis, focal segmental glomerulosclerosis (FSGS), ischemic, hypertrophic and solidified glomeruli, or global glomerulosclerosis (GGS). Data from animal models were compared to human reference values. Kidneys in TTRhRen mice, SHR and the nonclipped kidneys in 2K1C rats had no sign of hyalinosis, FSGS or GGS. Glomerulopathy in these groups was limited to variations in mesangial and capillary compartment volumes, with mild increases in collagen deposition. Histopathology in angiotensin II infused mice corresponded to mesangioproliferative glomerulonephritis, but not hypertensive glomerulosclerosis. The number of nephrons was significantly reduced in TTRhRen mice and SHR, but did not correlate with severity of glomerulopathy. The most substantial human-like glomerulosclerotic lesions, including FSGS, ischemic obsolescent glomeruli and GGS, were found in the clipped kidneys of 2K1C rats. The comparison of affected kidneys to healthy control in animals produces lesion values that are numerically impressive but correspond to mild damage if compared to humans. Animal studies should be standardized by employing the criteria and classifications established in human pathology to make experimental and human data fully comparable for comprehensive analysis and model improvements.

## Introduction

Hypertension and diabetes are major causes of cardiovascular morbidity and mortality worldwide [1] and leading causes of chronic kidney disease (CKD) [2]. Animal models of diseases are aimed to recapitulate the human conditions both at early stages and in advanced progressive disease [3]. This approach is well established in diabetes studies: while known rodent models reliably develop signs of early diabetic nephropathy, progression to CKD is usually absent [4,5]. To improve the quality of animal research, the Animal Models of Diabetic Complications Consortium was established in order to develop criteria for validating progressive diabetic nephropathy in animals. This critical assessment of models in diabetes research is an important constant stimulus for ongoing improvement [6–8].

Unfortunately, a criteria-based approach to pathologic lesions is not commonly found in experimental hypertension studies. A prolonged increase in systolic blood pressure (SBP) is the main criteria to consider a hypertension model useful for investigation, and more than fifty different models have been described [9–11]. As in diabetic models, hypertensive animals demonstrate only mild renal functional impairment [10,11]. Nevertheless these models are commonly used to study renoprotective therapy [12–15]. The inability to recapitulate progression to CKD in hypertensive models has led researchers to explore models less relevant to human hypertension, such as unilateral ureteral obstruction or subtotal nephrectomy [16–18]. This critical approach, as in diabetes research, is rather exclusive and only a few reviews have highlighted the negative features, problems, and inconsistency of current hypertensive models [3,19,20].

Obviously, an ideal animal model for hypertension research that reproduces all aspects of etiology and pathogenesis cannot be created, considering the pathophysiological heterogeneity and clinical diversity of the disease. Hypertensive models differ in the timing of onset and continuance, degree of SBP elevation, and severity of target organ damage, including CKD. Therefore, in order to verify the degree of reproducibility objectively, the resemblance of each model to human disease needs to be assessed. Urine and blood laboratory test results may be directly comparable between animals and humans because they employ similar methods. On the other hand, histopathology analysis may demonstrate critical disparities between human and animal studies in assessment approaches.

In human pathology, the severity of glomerulopathy is determined mostly by the value of global glomerulosclerosis (GGS) (**Table 1**), with the glomerular lesion score quantifying mild to severe mesangial expansion as a score of 1 or 2, progressing to segmental and global hyalinosis and/or sclerosis (score 3 and 4) [21,22]. Glomerulopathy in animal studies is poorly characterized and illustrated, with avoidance of terminology and classification in the major animal histopathology guides [23–25]. In animal pathology, glomerulopathy is estimated mostly by the glomerular lesion score (**Table 2**), which has varied definitions and is interpreted and scored subjectively for different variables such as mesangial expansion [26], mesangial matrix and glomerular fibrosis [15], mesangial matrix expansion and mesangial sclerosis/hyalinosis [12,27], glomerular fibrosis and mesangiolysis [28], mesangial cell proliferation, hyalinosis and sclerosis [16], mesangial cell proliferation and matrix expansion [29], and fibrinoid and/or crescent formation [30]. Other studies have analyzed ischemic, acute hypertensive injury, or segmental sclerosis [31], glomerular collapse, hypertrophy, and sclerosis [32], glomerular basement membrane (GBM) thickening, or mesangial hypertrophy and capillary occlusion [33]. Moreover, there are many formulas to quantify glomerular lesion score, with values ranging from 0–2 [16], 0–3 [27], 0–4 [34], 0–5 [35], 0–100 [36], 0–400 [12,37], or with yes/no binary variables [38] (**S1 Table**). Different criteria/parameters for semi-quantitative scoring and quantification make it difficult to properly validate the severity of hypertensive nephropathy in each study, and make comparison within animal studies, and with human data (**Tables 1 and 2**).

**Table 1:** Commonly accepted criteria for hypertensive glomerulopathy assessment enable quantification and comparison among human studies.

Author	Type of scoring	Values
Fogo et al. [39]: essential hypertension, 46 patients	FSGS, percentage GGS, percentage	~ 5% ~ 43±26%
Caetano et al. [40]: essential hypertension, 81 patients	GGS, percentage	~ 20±3%
Ballardie et al. [41]: IgA nephropathy with hypertension, 36 patients	% of glomerular area (score)	~ 1.6 (3.0 maximum)
Hughson et al. [42]: hypertensive nephropathy, 239 patients	% of glomerular area (score) GGS, percentage	~ 1.4 (4.0 maximum) ~ 29%
Marcantoni et al. [43]: hypertensive nephrosclerosis, 62 patients	GGS, percentage	~ 42±3%
Hill et al. [44]: essential hypertension, 30 patients	FSGS, percentage GGS, percentage	~ 24% ~ 18%
Ikee et al. [45]: IgA nephropathy with hypertension, 38 patients	GGS, percentage	~ 25%
Imakiire et al. [46]: essential hypertension, 31 patients	GGS, percentage	~ 21± 15%
Bige et al. [47]: chronic kidney disease, 58 patients	GGS, percentage	~ 13 (0–96) %
Liang et al. [48]: benign and malignant essential hypertension, 194 patients	FSGS, percentage GGS, percentage	~ 5–6% ~ 18–50%
Denic et al. [49]: normal aging, 1638 patients	GGS, percentage	~ 27%
Lee et al. [50]: IgA nephropathy with hypertension, 340 patients	FSGS, percentage GGS, percentage	~ 6% ~ 21%

The values are averaged from published data. FSGS—focal segmental glomerulosclerosis; GGS–global glomerulosclerosis.

**Table 2:** Variable parameters for hypertensive glomerulopathy assessment unable quantification and comparison among animal studies.

Model, author	Type of scoring	Values
**Angiotensin II infusion**		
Brand et al. [28]	% of glomerular area involved	Score ~ 0.64 (4.0*)
Gu et al. [51]	% of glomerular area involved	Score ~ 0.35 (5.0*)
Liao et al. [26]	% of mesangial matrix	~ 6% increase
Casare et al. [52]	% of glomerular area involved	Score ~ 0.5 (4.0*)
Polichnowski et al. [53]	% of ‘sclerotic’ glomeruli	~ 23% increase
**Spontaneously hypertensive rats**		
Gross et al. [13]	% of glomerular area involved	Score ~ 0.4 (4.0*)
Ishimitsu et al. [54]	% of glomerular area involved	Score ~ 19 (300*)
Raij et al. [55]	% of mesangial matrix	Score ~ 40 (400*)
Kohara et al. [56]	% of glomeruli, segmental lesions global lesions	~ 36% ~19%
**Goldblatt rats**		
Oboshi et al. [57]	For nonclipped kidney only: % of glomerular area involved	Score ~ 1.0 (2.0*)
Veniant et al. [12]	For clipped kidney: number of infarction in a field For nonclipped kidney: % of glomerular area involved	0–10 per animal Score ~ 19 (400*)
Konopka et al. [32]	For clipped kidney only: % of glomerular area involved	Score ~ 1.2 (4.0*)
Shao et al. [58]	For clipped kidney: % of PAS positive area For nonclipped kidney: % of PAS positive area	+3% +20%
Kobayashi et al. [59]	For clipped kidney: % of glomeruli with the score >2.0, For nonclipped kidney: % of glomeruli with the score >2.0,	19% Negligible
Steinmetz et al. [60]	For clipped kidney only: severity score	Normal

The score values are averaged values obtained from published data. *—maximum possible value; PAS–periodic acid-Schiff stain.

Optimization in analysis of pathologic variables is constantly improved by testing sensitivity, specificity, and predictive values for differences in clinical features and renoprotective treatment [41,47,48,61]. In human pathology, lesional variables are identified and optimized in order to predict progression, outcome, and response to therapy [21,42,43,62,63]. Unfortunately, in animal studies histopathologic features only indicate the presence of lesions but not the severity because there are no commonly accepted values and each study makes assumptions based on particular set of scale values. This inconsistency in assessment approach between human and animal studies makes meaningless comparisons between research studies and clinical trials.

In this study, we performed a detailed comparative analysis of glomerulopathy in different animal models of hypertension based on the criteria widely accepted in clinical pathology.

## Material and methods

All animal studies were approved by the University of Ottawa Animal Care Committee (Ottawa, ON, Canada) and conducted according to the guidelines of the Canadian Council on Animal Care. Experimental animals were housed and cared for in the Animal Care Facility at the University of Ottawa with free access to food and water. Animals were age-matched males (**Table 3)**.

**Table 3:** Characteristics of the animals at the end of the study.

	Mice			Rats		
	Control	Ang II	TTRhRen	Control	SHR	2K1C
Animals	Male FVB N = 6	Male FVB N = 8	Male TTRhRen N = 8	Male Wistar N = 8	Male SHR N = 12	Male Wistar N = 7
Age, weeks	24	22–24	24	22	25–27	22
Body weight, g	30.2 ± 1.3	26.4 ±1.6	31.3 ± 1.4	439 ± 12	382 ± 7	453 ± 29
Kidney weight, mg	202 ± 8	207 ± 8	216 ± 10	1265 ± 65	1228 ± 12	Nonclipped 1738 ± 84 Clipped 762 ± 73
SBP, mmHg	114 ± 3	168 ± 4	143 ± 3	130 ± 5	229 ± 5	263 ± 17
GFR μl/min	246 ± 23	230±70	231 ± 28	15 ± 3 L/kg/day	Not measured	9 ± 2 L/kg/day

Ang II–angiotensin II infused mice; TTRhRen–renin overexpressing mice; SHR–spontaneously hypertensive rats; 2K1C –two kidney, one clip rats; N–number of animals; SBP–systolic blood pressure; GFR–glomerular filtration rate.

### Angiotensin II -induced hypertension

Mini-osmotic pumps (Alzet,; Model 2004; Durect Corporation, Cupertina, CA, USA) containing sufficient angiotensin II (Ang II) for 4 weeks of drug delivery at a rate of 1000 ng/kg/min were surgically implanted subcutaneously. To implant the minipumps, mice were anesthetized with 3% isoflurane in oxygen and body temperature was maintained at 37^0^ C. Mice were given a single dose of the antibiotic and the analgesic prior to surgery. Control FVB mice underwent sham operation, omitting pump implantation. SBP was measured via tail-cuff plethysmography (Visitech Systems; Model BP2000; Apex, NC, USA), and the average was calculated from measurements obtained at the same period each day (5 preliminary, 10 actual SBP readings/day) and, after a 5-day training regimen (10 SBP readings/day), SBP measurements were obtained twice a week. At the time of euthanasia, mice were anesthetized (isoflurane), perfused with PBS and kidneys were excised, dissected, and immediately fixed in 4% paraformaldehyde. The body and kidney weight, plasma glucose level, albumin-to creatinine ratio, and glomerular filtration rate were measured and analysed as described [64].

### Renin overexpressing mice

To express human renin in mouse liver, a 3-kb region of the transthyretin gene promoter was cloned upstream of the human prorenin cDNA. To generate active human renin, a cleavage site for the ubiquitous protease furin was inserted at the juncture of the prosegment and the active renin molecule, resulting in prosegment removal by endogenous proteases in the secretory pathway of expressing cells [65]. Mice become chronically hypertensive by 8 weeks and develop cardiac hypertrophy by 20 months of age. Hemizygous TTRhRen mice and their wild-type littermates were used on an FVB background and studied at 24 wks of age. SBP was measured as in Ang II mice. At the time of euthanasia, mice were anesthetized (isoflurane), perfused with PBS and kidneys were excised, dissected, and immediately fixed in 4% paraformaldehyde. The body and kidney weight, plasma glucose level, albumin-to creatinine ratio, and glomerular filtration rate were measured and analysed as described [66].

### Spontaneously hypertensive rats

Adult (25–27 weeks old) male SHRs (Charles River, St-Constant, Quebec, Canada) were used. At the time of euthanasia, rats were anesthetized with sodium pentobarbital 60 mg/kg and the left carotid artery cannulated for SBP measurement using a pressure transducer (Harvard Apparatus, St Laurent, QC, Canada) and a digital data acquisition system (Model MP100, Biopac System, Harvard Apparatus). The body and kidney weight, plasma creatinine and urea level were measured and analysed as described [14].

### Two-kidney one-clip rats

Goldblatt two-kidney one-clip (2K1C) hypertension was induced in male Wistar rats. Animals were anaesthetized with tribromoethanol (50 mg/kg i.p.) and after a midline laparotomy, a silver clip with an internal diameter of 0.20 mm was placed around the left renal artery. Sham control rats were submitted to laparotomy only. SBP was measured before surgery and every three days over the period of treatments in awake animals by a tail-cuff method (MLT125R pulse transducer/pressure cuff coupled to the PowerLab 4/S analog-to-digital converter; ADInstruments, Castle Hill, Australia). The rats with SBP exceeding 160 mmHg were considered hypertensive. At the end of the experiment, the animals were weighed and killed after anesthesia with tribromoethanol (50 mg/kg i.p.). The body and kidney weight, tibia length, glomerular filtration rate were measured and analysed as described [67].

### Histology

The kidney tissues from these previously published studies [14,64,66,67] were used for current histopathologic analysis. Kidneys were fixed in in 10% neutral buffered zinc-formalin and embedded in paraffin. Midhilar 4-μm coronal cross sections of each kidney (4 per animal) were stained with hematoxylin and eosin, periodic acid-Schiff (PAS) and Masson trichrome. Microscopic investigations were performed in a masked manner on coded sections. Histopathology analysis was done by two pathologists with an interobserver agreement of 92%.

Images were acquired with a microscope Zeiss Imager A1 (Carl Zeiss, Oberkochen, Germany) equipped with Olympus camera DP73 (Olympus Canada, Richmond Hill, ON, Canada), and scans obtained with EVOS FLAuto2 scanner (Thermo Fisher Scientific, Carlsbad, CA, USA). Images were processed and analyzed by ImageJ software (National Institute of Health, Bethesda, MD, USA), and Aperio ImageScope v.12.3.3 software (Leica Biosystems, Wetzlar, Germany).

#### Glomerular histopathology and morphometry analysis

**Fig 1:**
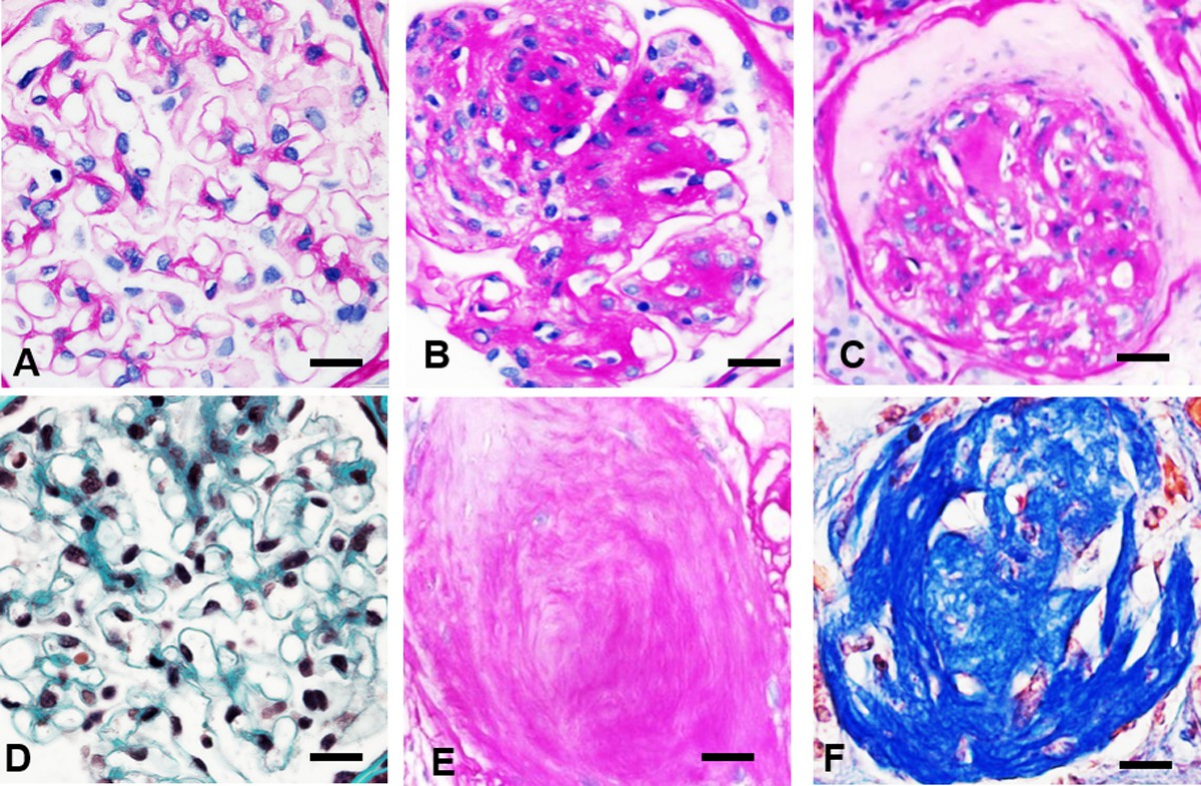
Criteria for hypertensive glomerulopathy in humans. **A, D**. Normal glomerulus. **B** In *solidified* glomeruli, mesangial widening results in prominent hyalinotic lesions and segmental sclerosis extending to Bowman’s capsule. Podocytes, mesangial and endothelial cells, capillary lumens gradually disappeared within expanding hyalinotic segments. **C** In *ischemic* glomeruli, the tuft retracted, capillary walls thickened while their lumens collapsed; hyalinosis and segmental sclerosis are less prominent. **E, F** Either solidified or ischemic glomeruli progress into the globally sclerotic lesion–acellular collagenous tufts. **A—C, E**–periodic acid–Schiff; **D, F**–Masson trichrome staining. x600.

The general histopathology assessment was based on finding *hypertrophic*, *solidified* and *ischemic obsolescent* glomeruli, which are key features to identify hypertensive glomerulopathy in humans [68,69]. *Solidified* and *ischemic* glomeruli exhibit gradual loss of podocytes, mesangial cells, endothelial cells, and capillary lumens that are replaced by expanding PAS-positive hyalinotic substance containing mature collagen. Each type progresses into GGS (**Fig 1**). The images representing human hypertensive nephropathy were obtained from human renal biopsies used in our published study [70].

Instead of opinion-based, widely defined glomerular lesion score used in animal studies (**Tables 1 and S1**), we applied the pathologic variables for human glomerulopathy [21,62,71] **(S2–S4 Tables)**. In human studies, lesional variables are semi-quantified with distribution and/or severity descriptors because it is more practical for scoring small needle biopsies. Since whole kidneys are available in animals, each lesional variable was quantified with image analysis used in our previous studies [17,64,72]. Five lesional descriptors were quantified: mesangial expansion, mesangial cellularity, hyalinosis, FSGS, and GGS.

In each kidney, a minimum of 20 glomeruli (range, 20 to 30, i.e. 400 to 600 for each group) with apparent *macula densa* and arterioles were imaged in the outer cortex at a magnification x400 on PAS-stained paraffin sections. The minimal convex polygons circumscribing the capillary tufts of the glomeruli were manually traced, duplicated into the area of interest and measured as the glomerular area (GLA). The mesangial area (MEA) was defined as the PAS-positive area inside GLA, and quantified using threshold adjustment and segmentation. On trichrome stained sections, the same procedure was applied to measure glomerular sclerosis area that was quantified as a percentage of GLA [15,26]. GGS was considered if glomerular sclerosis area was >50% of GLA. Mesangial cellularity (MEC) was measured as the number of nuclei of mesangial cells in MEA. To identify *ischemic* and *hypertrophic* glomeruli, the mean glomerular volume (GLV) was calculated from the GLA with the following formula

$$GLV={GLA}^{1.5}\times \frac{\beta }{\kappa }$$ (1)

where β is a dimensionless shape coefficient (1.38 for spheres), and κ is a size distribution coefficient that corrects for variation in the glomerular size. We used κ = 1.01 as in other studies [42]. The mesangial volume (MEV) was determined according to the basic stereological assumption that the areal fraction A_A_ is equal to the volume fraction V_V_ [73]:

$$if\frac{GLA}{MEA}=\frac{GLV}{MEV},\;\mathrm{then}\;MEV=\frac{MEA}{GLA}\times GLV$$ (2)

The respective glomerular capillary volume (GCV) was calculated as

GCV=GLV−MEV (3)

Numerical density (number of tufts per unit volume) of glomeruli (N_v_) was estimated as

NV=NA÷√4GLAπ (4)

where N_A_ is the number of tufts per field of view, and A is the area of the field of view. The counting was done in 10 to 20 fields for each kidney section (i.e. 300–400 measurements per group) in the cortex at a magnification x100 on PAS-stained sections.

Frequency of glomerular hyalinosis, FSGS and GGS was estimated in whole section scans, in a range of 2400 to 3600 glomeruli per group. Discrimination between sclerosis and hyalinization was based on the widely accepted definition: sclerotic areas were stained red by PAS staining and blue or green by Masson-trichrome staining, and hyalinotic areas were stained red by both PAS staining and Masson-trichrome staining [74]. Experimental data was compared with reference values in humans obtained in clinical studies (**Table 1**).

## Statistical analysis

Distribution was assessed by Kolmogorov-Smirnov, D’Agostino & Pearson and Shapiro-Wilk tests. P < 0.01 was regarded as significant. Data is represented as means ± SEM. Differences between two groups were recognized by unpaired t-test with Welch’s correction. For more than two experimental groups, one-way analysis of variance was applied, followed by post hoc comparisons (t-test). The values of the glomerular volume demonstrated non-Gaussian distribution and outliers therefore were compared with the Mann-Whitney test. Outliers were identified by the combined robust regression and outlier removal method. Frequency of hyalynosis was analysed with the chi-square test. Prism GraphPad software version 8.4.0 (GraphPad Software, San Diego, CA, USA) was used for statistical analyses.

## Results

There were no differences between control and hypertensive animals in body and kidney weights. As previously reported, all models developed stable hypertension and mild impairment in renal function [14,64,66,67] (**Table 3**).

### Glomerulopathy in the mouse models

**Fig 2:**
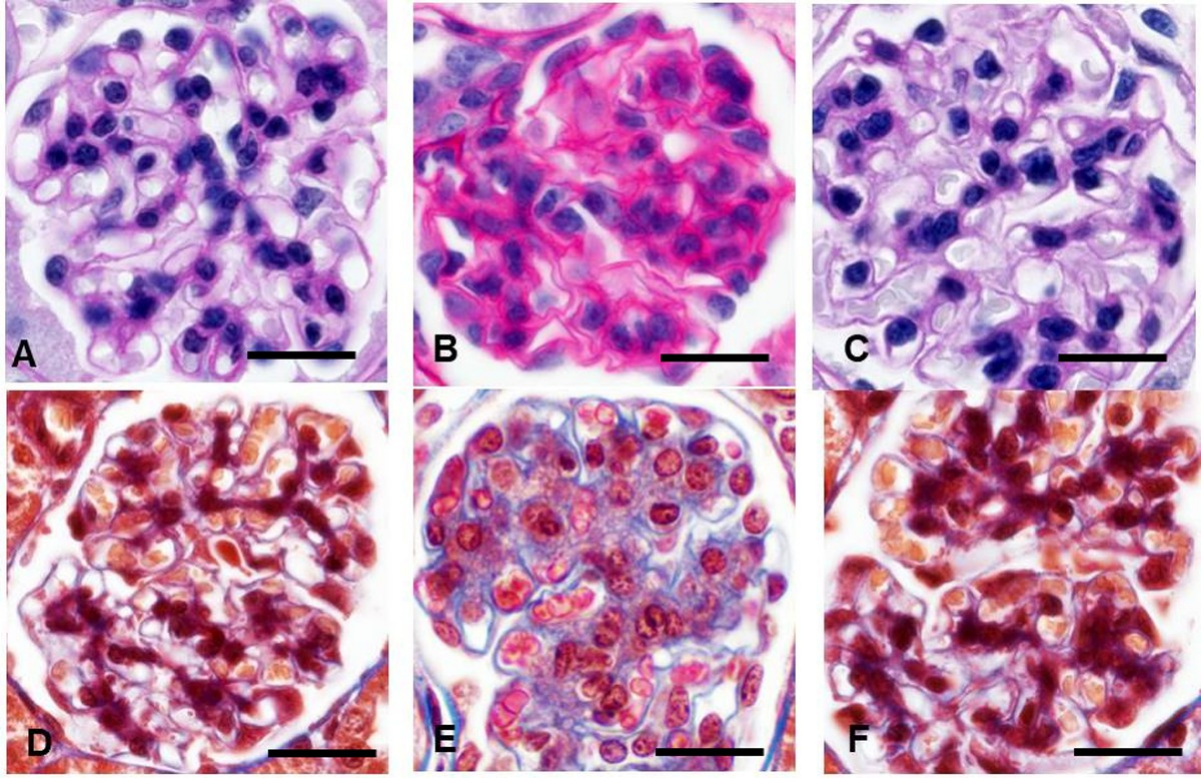
Representative images of hypertensive glomerulopathy in mouse models. **A, D** Normal mouse glomerulus. **B, E** Angiotensin II infused mice. Diminished glomeruli contain abundant mesangial cells, expanded mesangial matrix, and reduced capillary lumens. Prominent mesangial expansion has mild fibrotic component: Collagen staining increased along the glomerular basement membrane. **C, F** Glomeruli in the renin overexpressing mice appear unremarkable. Hyalinosis and focal segmental glomerulosclerosis was absent in both models. **A-C**–periodic acid-Schiff; **D-F**—Masson trichrome staining. x600.

**Fig 3:**
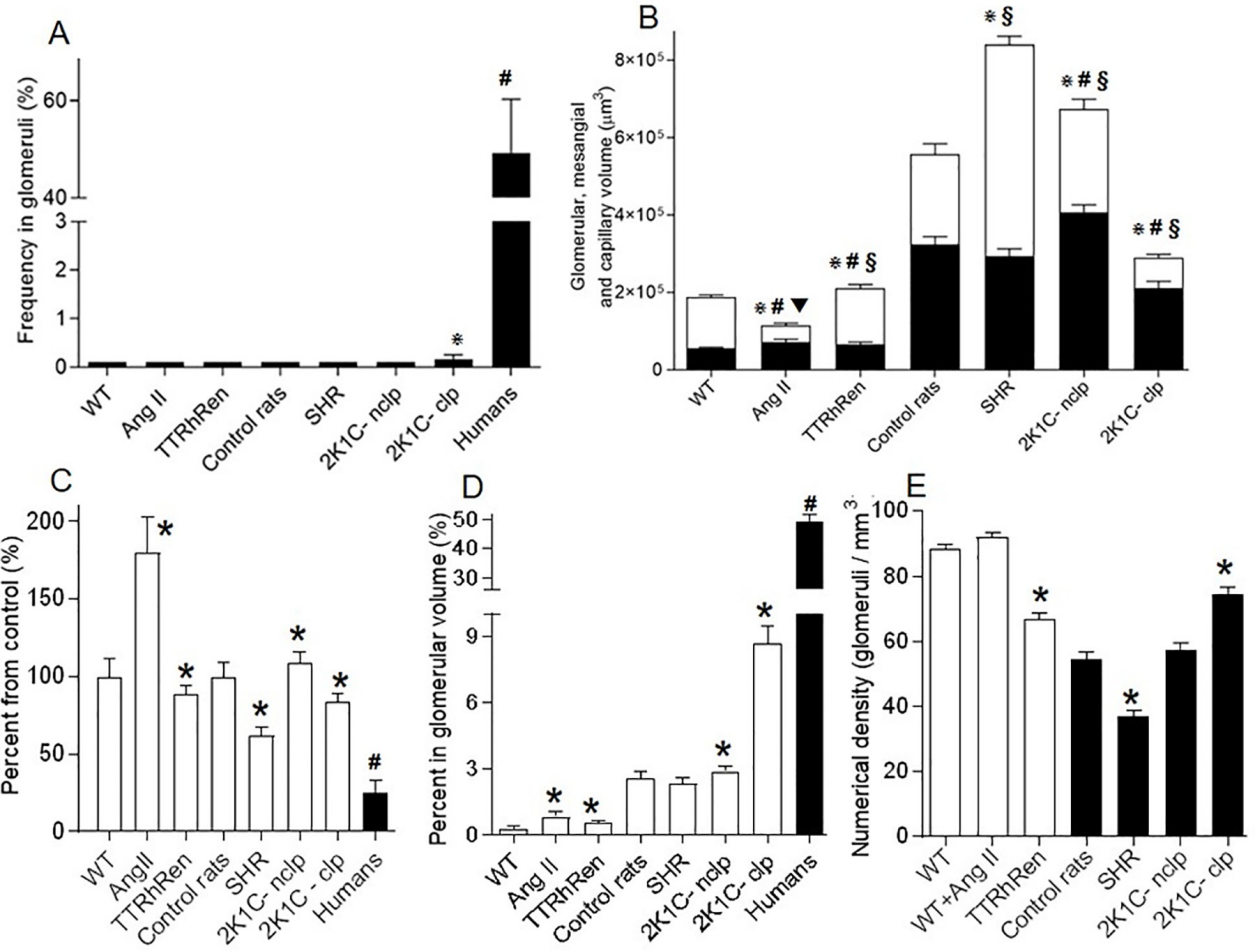
Glomerular lesions in hypertensive animal models were much lower compared to humans. A Frequency of hyalinosis/focal segmental glomerulosclerosis is presented as the percentage of positively stained tufts. Among samples, only the clipped kidney (2K1C-clp) demonstrated lesions (⋇ P<0.01 vs control) that remarkably lower than human values (# P < 0.0001 vs 2K1C-clp). B Morphometric data of the mesangial, capillary and glomerular volumes. The whole bar represents the glomerular volume as the sum of the mesangial (black part) and capillary (white part) volumes. P < 0.01 vs control for mesangium (⋇), capillary (#), and glomerular (§) volumes respectively. C Mesangial cellularity markedly increased in angiotensin II infused mice (Ang II) and decreased in spontaneously hypertensive rats (SHR) (⋇ P < 0.001 vs control) but still far from hypocellularity observed in human glomerulosclerosis (# P < 0.001 vs SHR). D The collagen-positive area in glomeruli. P < 0.001 vs control (⋇). TTRhRen—renin overexpressing mice; 2K1C-nclp—non-clipped kidneys. Human data are reference values from Table 1. E Genetic models of hypertension demonstrated lower number of nephrons. P < 0.01 vs control (⋇). Each column consists of mean ± SEM.

*Ischemic* and *solidified* glomeruli were not found in either of the two mouse models of hypertension. The key features of hyalinosis, FSGS and GGS were also absent in both models (**Figs 2 and 3A**). The most significant structural changes were observed in Ang II infused mice. Cortical tufts were reduced in size with no sign of *ischemic* glomeruli and visible collagen increases in a GBM-like pattern (**Fig 2**). Ang II infusion caused a 39% reduction in GLV due to severe decline in the capillary compartment (-68%) while the mesangium was expanded (+29%), contained almost double the number of mesangial cells (+93%), and tripled collagen volume (+196%) (**Figs 3B–3D and S1**).

Minor variations were detected in TTRhRen mice (**Fig 2).** The mean GLV was slightly enlarged (+12%) with increased GCV (+10%) and MEV (+18%) but fewer mesangial cells (-12%) and more collagen (+98%). The increase in glomerular collagen (up to 3-fold) was statistically significant and could be considered very high if compared to the control value that was close to zero. However, the enhancement was small compared to the 19-fold increase observed in humans (**Fig 3B–3D).** *Hypertrophic* glomeruli were detected on the glomerular volume histogram as 10% fraction of ~3-fold larger tufts (**S1 Fig**).

### Glomerulopathy in the rat models

**Fig 4:**
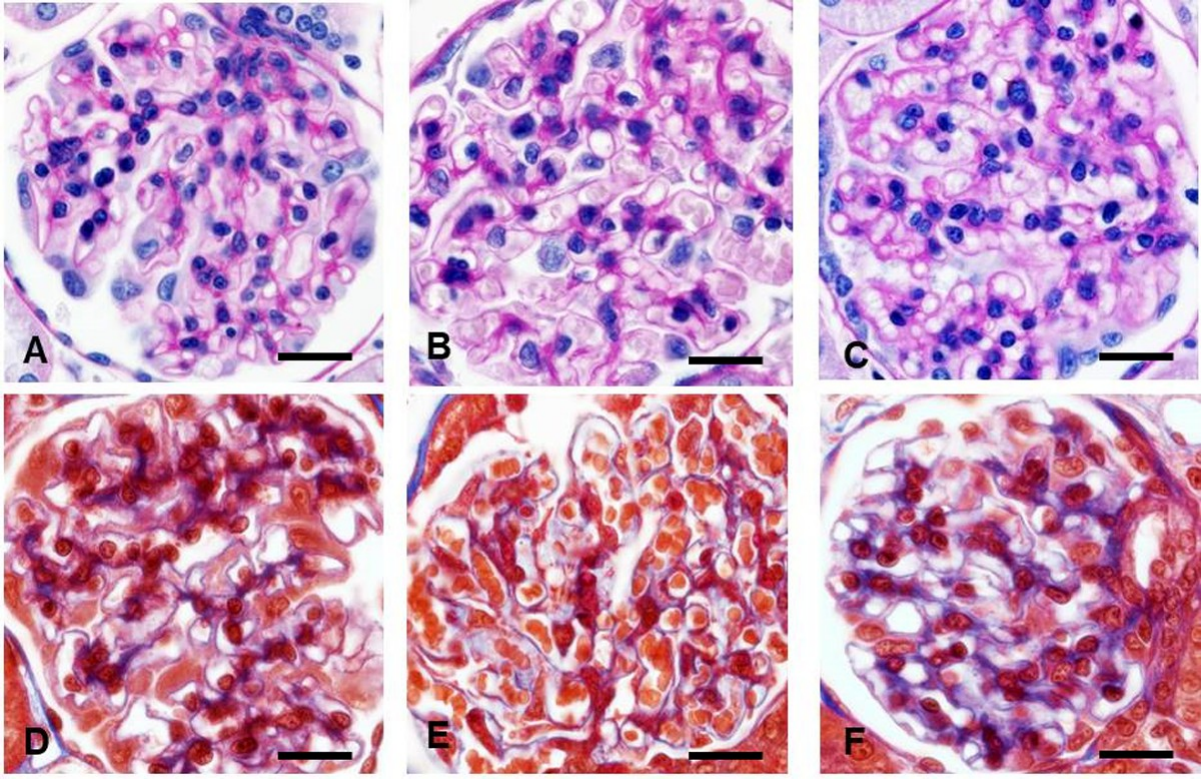
Representative images of hypertensive glomerulopathy in rats. **A, D** Control rats. **B, E** Spontaneously hypertensive rats. *Hypertrophic* glomeruli are distinguished with numerous enlarged capillary lumens. **C, F** The nonclipped kidney. *Hypertrophic* glomeruli had no visible lesions except enlarged capillary loops and more collagen that distributed along the glomerular basement membrane. Hyalinosis and segmental sclerosis was absent in all models. **A-C**—periodic acid-Schiff; **D-F** Masson trichrome staining. x600.

In the SHR model, *ischemic* and *solidified* glomeruli were not found. Hyalinosis, FSGS and GGS were not detected (**Figs 3A and 4**). The 56% fraction of glomeruli was *hypertrophic* (**S2 Fig**). The mean GLV was noticeably larger (+51%), predominantly for the capillary compartment (+134%), while MEV was slightly reduced (-10%). MEC was greatly diminished (-38%), and collagen positive area was unchanged (**Figs 3B–3D and** **4**).

In the non-clipped kidney from 2K1C rats, *solidified* and *ischemic* glomeruli were not detected and the incidence of hyalinosis was extremely low (~ 0.08%), while FSGS and GGS were absent (**Figs 3A,** **4C** and **4F**). *Hypertrophic* glomeruli (34% of total) demonstrated enlarged GLV (+21%), with +15%, +26%, and +9% enhancement in GCV, MEV and MEC respectively. Collagen was mildly expanded (+12%) and appeared as a thickened GBM-like pattern (**Figs 3B–3D, 4C, 4F and 6A; S2 Fig**).

Glomerulopathy was most distinctive in clipped kidneys with some similarities to human lesions, although differ substantially. All aforementioned models demonstrated relatively even involvement of the cortex. The histopathologic lesions in the ischemic kidney were extremely irregular occupying from 5 to 80% of the renal cortex (**S3 Fig)**. *Ischemic* glomeruli represented an 84% fraction of measured tufts (**S2 Fig)**, while *solidified* glomeruli were not found. The frequency of hyalinosis/FSGS in the *ischemic* glomeruli was very low (~ 0.5%), compared to control rats, and GGS was absent (**Figs 3A and** **5**). These lesions were verified with corresponding morphometry data. Glomeruli were reduced in volume (-49%) due to severely attenuated GCV (-68%) and MEV (-35%). MEC was diminished (-16%), while collagen was expanded by 3.4-fold (**Figs 3B–3D and S2)**.

**Fig 5:**
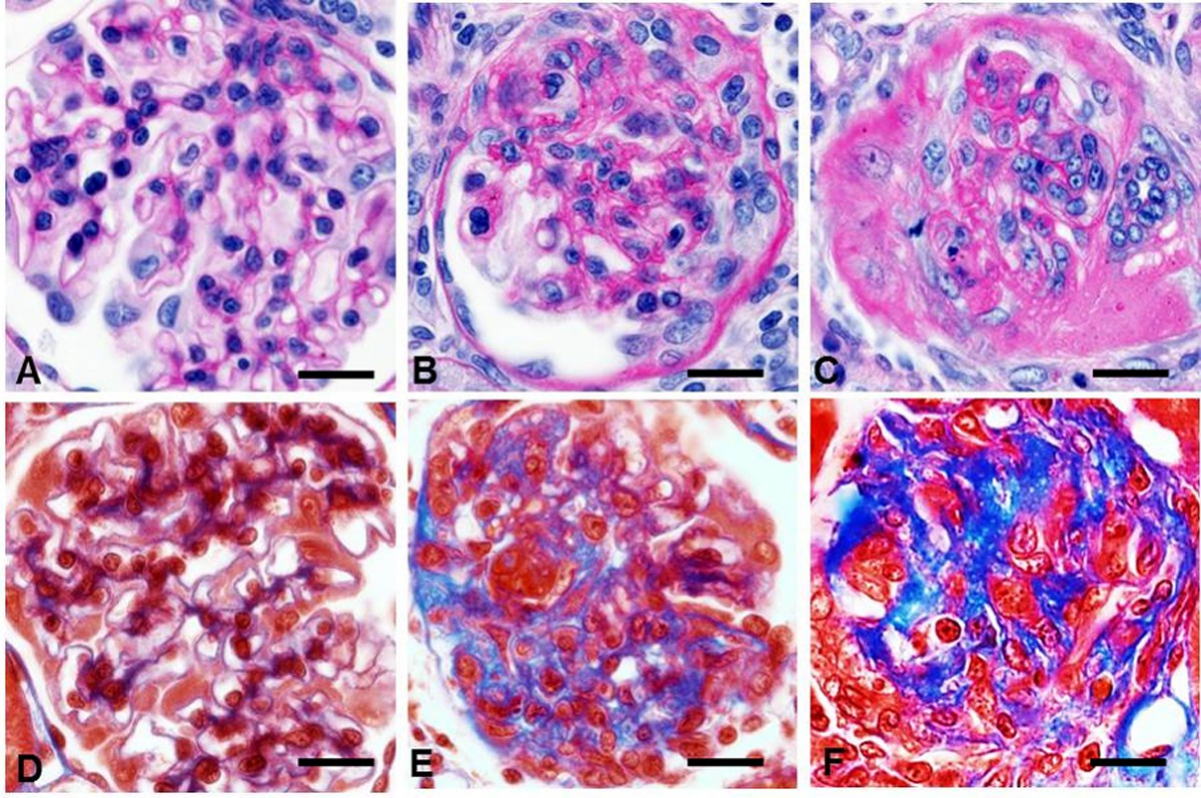
Hypertensive glomerulopathy in the clipped kidney resembled human glomerulosclerosis. **A, D** Control rats. **B, C, E, F** Tufts showed features of the *ischemic glomeruli*, including reduced capillary lumens, retracted tufts, narrowed Bowman’s spaces, PAS filled mesangium with small collagen amount. **A-C—**periodic acid-Schiff; **D-F**—Masson trichrome staining. x600.

These data indicate that hypertensive glomerulopathy in rats was minimal in SHR and the nonclipped kidney with mild changes in mesangial cellularity, mild fibrosis, and absence of hyalinosis or FSGS. The clipped kidney developed *ischemic* glomeruli, but the prevalence of hyalinosis/FSGS was very low, while GGS was absent. A marked increased (3.4-fold) in tuft collagen could be considered high if compared to control values, however this enhancement is only 15–20% of the severity of fibrosis observed in humans (**Table 1**).

### Number of nephrons

A significant (-25%) decline in Nv was detected in TTRhRen mice (**Fig 3E**). Since there is no difference in kidney size between TTRhRen and control mice, this may indicate that TTRhRen mice have 25% less glomeruli (nephrons). Nv was also markedly (-32%) reduced in SHR (**Fig 3E)**. Since there is no difference in kidney weight between SHR and control rats, this may indicate that SHR have 32% less glomeruli (nephrons). The reduced number of nephrons did not affect the severity of glomerulopathy in these models. In the clipped kidney Nv was enhanced (+37%) as a consequence of a significant reduction in the parenchymal volume.

## Discussion

Research into the vast majority of renal diseases is well supported by renal biopsies that enable a full spectrum of histopathological, genetic, and biochemical analysis. In sharp contrast, enormous numbers of patients with essential hypertension (~30% of the world’s adult population [1]) account for only solitary few studies employing renal biopsies over the last 20 years [48,75]. Therefore, animal models of hypertension are of critical importance for studying the renal pathology of hypertensive disease. At the same time, there is a significant discrepancy between assessment methodologies for hypertensive glomerulopathy in clinical and experimental studies.

In most animal studies, glomerular lesions are averaged in one score (**S1 Table**) while in humans they are classified into 5 to 11 independent pathologic variables each having their own semi-quantitative values and definitions for severity/distribution [21,71,76] (**S2–S4 Tables**). *Mesangial expansion*, unlike in animal studies [27,34], is an independent variable distinct from *hyalinosis*. *Mesangial expansion* results from excessive production of extracellular matrix and deposition of immunoglobulins (Ig) or immune complexes, that is attributed mainly to glomerulonephritis (GN) (secondary membranous, complement 3 (C3), fibrillary), monoclonal Ig deposition and dense-deposit disease [69]. *Hyalinosis* is a very important histopathologic feature indicating irreversible injury to the permeability barrier resulting in serum protein leakage and deposition of plasma proteins. *Mesangial sclerosis* in animal research is utilized equally to *mesangial expansion* and *hyalinosis* (**S1 Table**), but the human pathology operates with the feature of FSGS, subdivided, in turn, into five types [62] (**S4 Table**) that are not used in animal studies. *Mesangial cellularity*, unlike in animal studies [16,29], is a pathological variable independent from *mesangial expansion*, *hyalinosis* or FSGS and indicates GN–IgA nephropathy, lupus nephritis, rheumatoid arthritis-related nephropathy [69]. In animal studies the GBM *folding* is estimated together with GBM *thickening* [34,55]. In human pathology *GBM folding* characterizes collapsing glomerulopathy [77], while *GBM thickening* is a diagnostic feature of membranous nephropathy, dense deposit disease, C3 and diabetic nephropathy [69]. In animal studies *crescents* and *adhesion* to the Bowman’s capsule are analyzed as components of glomerular lesion score [30]. In human pathology they are independent pathological variables: *crescents* are an important diagnostic feature of GN [78] while *adhesions* could appear in many renal diseases [69]. Moreover, it has been shown in human pathology that hypertensive glomerulopathy occurs through three types of glomerular remodeling–*hypertrophic*, *solidified*, *and ischemic obsolescence* [42,68,69]. However, animal studies do not use this approach, with a few exceptions [32,79].

Indeed, when analyzed using human histopathology criteria, mouse models have minimal similarity with human hypertensive glomerulopathy. TTRhRen mice had no visible glomerular lesions. Light mesangial expansion and hypocellularity were detected only with morphometry, and the increase in the collagen volume was detected only by image analysis. The increased glomerular collagen and other lesions are invariably verified in hypertensive animals but the implementation of this fact should be more critical. In animal studies, the severity of glomerulopathy is scaled from the lowest (zero) point. Therefore a ‘high’ score, for example 0.64 (**Table 2, line 1**) indicates a 64-fold increase in severity from ~ 0.01 control. That value, impressive in both absolute value and statistical significance, in fact represents only mild lesions involving ~16% of the glomerular area. Some studies described periglomerular infiltration by macrophages and T-lymphocytes [80] that characterizes GN [81] and should be thoroughly investigated. In more advanced double transgenic Tsukuba mice GGS was increased to only 6% from control [74] but raised drastically if high salt diet was added [82]. In our experiments, GN was found in Ang II infused mice, where mesangial expansion was obvious, although a 3-fold increase in collagen was detected by image analysis and sometimes visible as capillary wall thickening. Those signs together with the increased number of mesangial cells corresponded to mesangioproliferative GN [69]. Decreased GLV found in this model is also similar to declined GLV in human GN [42]. Our data of increased mesangial cellularity in Ang II infused mice is difficult to compare with other studies since mesangial expansion has been scored or quantified in many studies, but mesangial cellularity has rarely been precisely analyzed, although mesangial cell proliferation was often considered in the scores (**S1 Table**). Mesangioproliferative GN in Ang II infused mice could explain the effective blood pressure lowering by interference in the inflammatory cascade [83], as was shown in humans [84]. Low degrees of glomerulosclerosis have been demonstrated in most Ang II infusion studies (**Table 2**). Even long term infusion complicated by nephrectomy reached a small score value of 0.35 out of 3 maximum [85].

The SHR is the most widely used animal model of essential hypertension and accompanying cardiac and renal involvement yet it has no features of CKD [3,9,10]. On the human scale, SHR demonstrated no lesions even though many glomeruli were *hypertrophic*. In parallel, other studies have shown unaltered expression of nephrin, nestin, desmin, perlecan, transforming growth factor-β1, or alfa-actin, and unchanged proliferative, apoptotic and oxidative stress markers [86,87]. Only electron microscopy revealed significantly reduced numbers of podocytes, and the size of endothelial fenestrae but not GBM or podocyte lesions [86]. There have been numerous attempts to increase kidney lesion severity in SHR by adding nephrectomy [29], renal ablation [56], nitric oxide synthase inhibition [14,87], deoxycorticosterone acetate–salt [88].

Our results are similar to previous reports that found larger glomeruli in SHR [89–92], but some studies detected no change [93,94]. Our data are in agreement with studies that show decreased number of nephrons in SHR [90,93], although some disagree [94]. This discrepancy might be due to different morphometry technique that results in ~14 fold difference in GLV values in control animals [95]. Taken together, the data suggest that SHR possess *hypertrophic* glomeruli because the nephron number is reduced innately, and is not a consequence of hypertensive glomerulopathy since *hypertrophic* glomeruli did not progress to *solidified* or *ischemic* forms. It is rather a structural adaptation than hypertensive lesions. Nevertheless, aged SHR could be more representative: a unique study on serial sections revealed the *hypertrophic*, *solidified* and *ischemic obsolescent* types of glomerular transformation [79] and advanced glomerulopathy [92]. The time of exposure and age are two crucial independent variables in pathogenesis of any disease. It is known that prevalence of hypertension among those ~40 years of age is ~37%, but for ≥75 years of age, prevalence is ~80%, while severity of target organ damage, complications and mortality drastically increased [96]. Mild alterations found in studied models could result from significantly shorter exposure time and younger age, compared to clinical hypertension. Interestingly, despite longer congenital hypertension and decreased number of nephrons in TTRhRen and SHR groups, histopathology was less prominent than in Ang II and 2K1C groups. Presumably, during early ontogenesis, the time could play a protective role by enabling adaptation mechanisms. Models of prolonged hypertension in aged animals could more closely resemble human disease. Therefore it is also important to apply the same appropriate criteria to distinguish aging and hypertensive pathology.

It has been suggested that a reduced number of nephrons is an important factor contributing to systemic hypertension and glomerular injury [69]. Surprisingly, TTRhRen mice demonstrated striking similarity to SHR in nephron number, and glomeruli were *hypertrophic*. Interestingly, lower nephron number and *hypertrophic* tufts characterize genetic hypertensive models, such as Munich-Wistar-Frömter and Prague rats [97,98], and our SHR and TTRhRen mice as well, which may indicate common genetic and environmental regulators of nephrogenesis.

The 2K1C model enables analysis of the kidney in two different pathogenic modules: the ischemic renin-producing kidney is protected from the high SBP; the contralateral kidney is overflowed, renin-suppressed and exposed to the high SBP. This model stood apart from other models by demonstrating profound irregularity in the extent of the cortical area involved in the compromised kidney (**S3 Fig**). Unpredictable irregularity of the hypertension course in 2K1C rats is well documented—up to 50% of operated animals could not maintain stable high SBP [12]. In contrast, about 10 to 50% develop malignant hypertension and die within the first weeks after surgery [60,99,100]. Some studies have found an immediate increase during the first week after clipping which was followed by a gradual decrease to baseline blood pressure [15,57]. The clipped kidney weight, an indicator of ischemia severity, was found to vary from 10 to 90% [32,38,57]. In the clipped kidney, many studies identified severe glomerulosclerosis [15,32,57], while others found only mild glomerulopathy that was less than in the nonclipped kidney [58,59,99], or even absent [60,99,101]. Significant variation in plasma renin activity has also been observed [102].

Analysis of the literature identifies several causes of variability. First, and most importantly, is the surgical technique that is unable to provide a consistent and precise degree of stenosis. Experimental induction of renal artery stenosis is traditionally achieved by U-shaped clips which do not guarantee the degree of stenosis and corresponding hypertension. The procedure requires not a simple clipping but a precise microsurgical tissue dissection and clip placement because a very small gradient (20 μm in rats and 10 μm in mice) is responsible for either successful hypertension or failure in the form of normotension or renal infarct. Indeed, animals subjected to graduated stenosis exhibit increased SBP and plasma renin activity precisely attributed to the degree of narrowing [103–106]. Therefore some studies have tried to increase effectiveness by using a cylindrical probe [32], special clamp [103], spiral [107], intra-arterial coil [9], and specially designed clip [106]. A second important reason for data variation is a wide SBP baseline to consider animals being hypertensive, ranging from >120 [58], >140 [38,57,100], >150 [27], >160 [60], or >170 [12] mm Hg. Third, the arterial wall and lumen at the clip side could be compromised by scar formation, or muscular and endothelial proliferative response but analysis of this issue is absent. Therefore the results of 2K1C studies should be analyzed with these factors taken into consideration.

In the nonclipped kidney, similar to our study, mild mesangial expansion and fibrosis in hypertrophied glomeruli has been shown [12,15,27,58]. Moreover, many studies claim no glomerular lesions [100,101]. Few studies declare severe glomerulosclerosis but it is poorly documented [57,59]. Some studies have found glomerulopathy associated with tubulointerstitial lesions, therefore the possibility of secondary glomerular involvement should be clarified. Unfortunately, uncertain degrees of stenosis and criteria for renal histopathological assessment have created a situation where no single study has correlated the damage severity in the clipped kidney to the nonclipped kidney.

**Fig 6:**
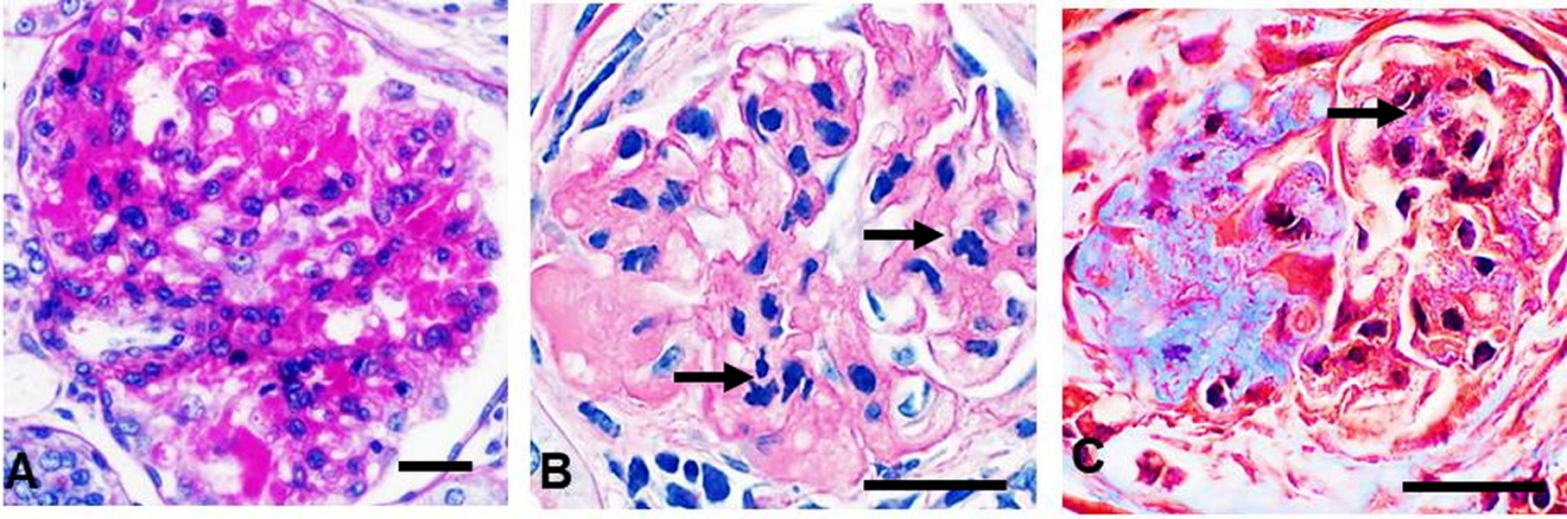
Detailed histopathological features in hypertensive animals. **A** Only few solidified glomeruli with hyalinosis were found through all scanned sections in the non-clipped kidneys. **B, C** Classical human-like focal segmental glomerulosclerosis in solidified glomeruli was more frequently detected in renin overexpressing mice with streptozotocin-induced diabetes [92]. Total karyorrhexis (arrows) and mature collagen in globally sclerotic glomeruli was easily found reflecting irreversible glomerular damage. **A, B—**periodic acid-Schiff; **C**—Masson trichrome staining. x600.

Characteristic features of human hypertensive glomerulopathy were found only in the clipped kidney in the form of *ischemic* glomeruli, FSGS and GGS (**Fig 5**). Nevertheless, the low frequency of hyalinosis and degree of FSGS (**Fig 3A and 3D**), and absence of those features in other studied models indicate significant resistance of the glomerular permeability barriers in rodents to structural lesions caused by hypertension alone but the mechanisms of this resistance are not known. In human primary hypertension, systemic and local vascular permeability increased [108]. A similar rise in glomerular albumin permeability was evident in SHR, 2K1C, and Ang II infusion models [91,109] but did not result in hyalinosis. Evidently, as shown in our previous studies, exposure to concomitant factors, e.g. hyperglycemia (streptozotocin—induced diabetes) was sufficient to cause valuable glomerular hyalinosis and FSGS in TTRhRen mice [110] (**Fig 6B and 6C).**

Importantly, fibrotic acellularity in FSGS is achieved through non-inflammatory lesions, and not neutrophil or monocyte/macrophage reaction [111]. In patients with hypertensive FSGS, various degrees of immune complex deposition were identified, including IgM, IgA, C3, C1q [75,112,113] and C3c, C5b9 in the malignant form [114] indicating IgM-mediated activation of the complement system. Noticeably, similar depositions have not been verified or characterised in any of the numerous hypertensive models [9–11]. From more than fifty animal models of hypertension, only the renal ablation and Munich-Wistar-Fromter rats are considered valuable to investigate FSGS [18] although ‘intensive glomerulosclerosis’ was declared in many studies. Absence of hyalinosis/FSGS in animal models unable to investigate the pathogenesis of glomerulopathy, and uncertain criteria of the assessment also hamper these studies.

Some limitations of our study should be acknowledged. SBP was measured by tail cuff plethysmography but not radio telemetry that is generally considered the gold standard. In addition, only males were used in all experiments.

### Conclusions

- Animal models are highly intended to recapitulate human diseases, therefore the variety of hypertensive models require to enforce uniformity for criteria, terminology and classification to compare animal and human histopathology.
- Each pathological variable, including glomerular cellularity, mesangial expansion, hyalinosis, focal segmental glomerulosclerosis, and global glomerulosclerosis must be analyzed separately. The usage of ‘integrated scores’ should not be advised. It is also important to eliminate glomerulonephritis.
- It is necessary to develop an international consensus classification for hypertensive glomerulopathy that will enable a good interobserver reproducibility and provide effective comparison among animal models and with humans.
- The most reasonable way to identify optimal models is to compare histopathological lesions not to healthy control animals but to the values that are identified for clinical stages of the disease. The hypertensive model should be considered effective if the reproducible identification of the following measures is achieved—greater than 40% frequency of hyalinosis and FSGS; greater than 20% frequency of GGS.
- Such efforts are critical to provide meaningful comparisons between research studies and clinical trials since inadequate animal data impair clinical translation.

## Supporting information

S1 Fig The glomerular volume distribution in mouse models. Control mice demonstrated the normal distribution curve (P>0.001). In the angiotensin II infused mice the decreased mean glomerular volume explains the left shifted curve (P<0.001). In the renin overexpressing mice the right asymmetry represents a pool of hypertrophic glomeruli (P<0.0001).
(PDF)

S2 Fig The glomerular volume distribution in rat models. The normal distribution curve characterises control rats (P>0.0001). The asymmetric right shifted curve and outliers in spontaneously hypertensive rats is the results of the increased glomerular volume (P<0.0001). The augmented glomerular volume in the nonclipped kidneys results in a normal distribution (P>0.001). The distribution curve in the clipped kidney is sharply left shifted because of numerous ischemic collapsed glomeruli (P<0.0001).
(PDF)

S3 Fig Variable appearance of histopathological lesions in the renal cortex of the clipped kidney. Extent of involvement of the cortical area varies among animals. They could be minimal (3–5% of the renal cortex, upper image, animal #0524) or occupy the entire cortex (60–80% of the renal cortex, lower image, animal # 0341) Scale 500 μm, x25, hematoxylin-eosin.
(PDF)

S1 Table Diversity of the glomerulosclerosis score in animal studies. (PDF)

S2 Table Semi-quantitative values or definitions of severity/distribution for pathological variables in the Oxford classification of IgA nephropathy. (PDF)

S3 Table Semi-quantitative values or definitions of severity/distribution for pathological variables in the classification of diabetic nephropathy. (PDF)

S4 Table Semi-quantitative values or definitions of severity/distribution in the classification of focal segmental glomerulosclerosis. (PDF)
