# Supplementary material for: Comparative analysis of hypertensive nephrosclerosis in animal models of hypertension and its relevance to human pathology. Glomerulopathy
Source: PLoS One. 2022 Feb 17;17(2):e0264136. doi: 10.1371/journal.pone.0264136 (PMC8853553; doi:10.1371/journal.pone.0264136)
Supplement: S1 Fig — Control mice demonstrated the normal distribution curve (P>0.001). In the angiotensin II infused mice the decreased mean glomerular volume explains the left shifted curve (P<0.001). In the renin overexpressing mice the right asymmetry represents a pool of hypertrophic glomeruli (P<0.0001). (PDF) [file pone.0264136.s001.pdf]

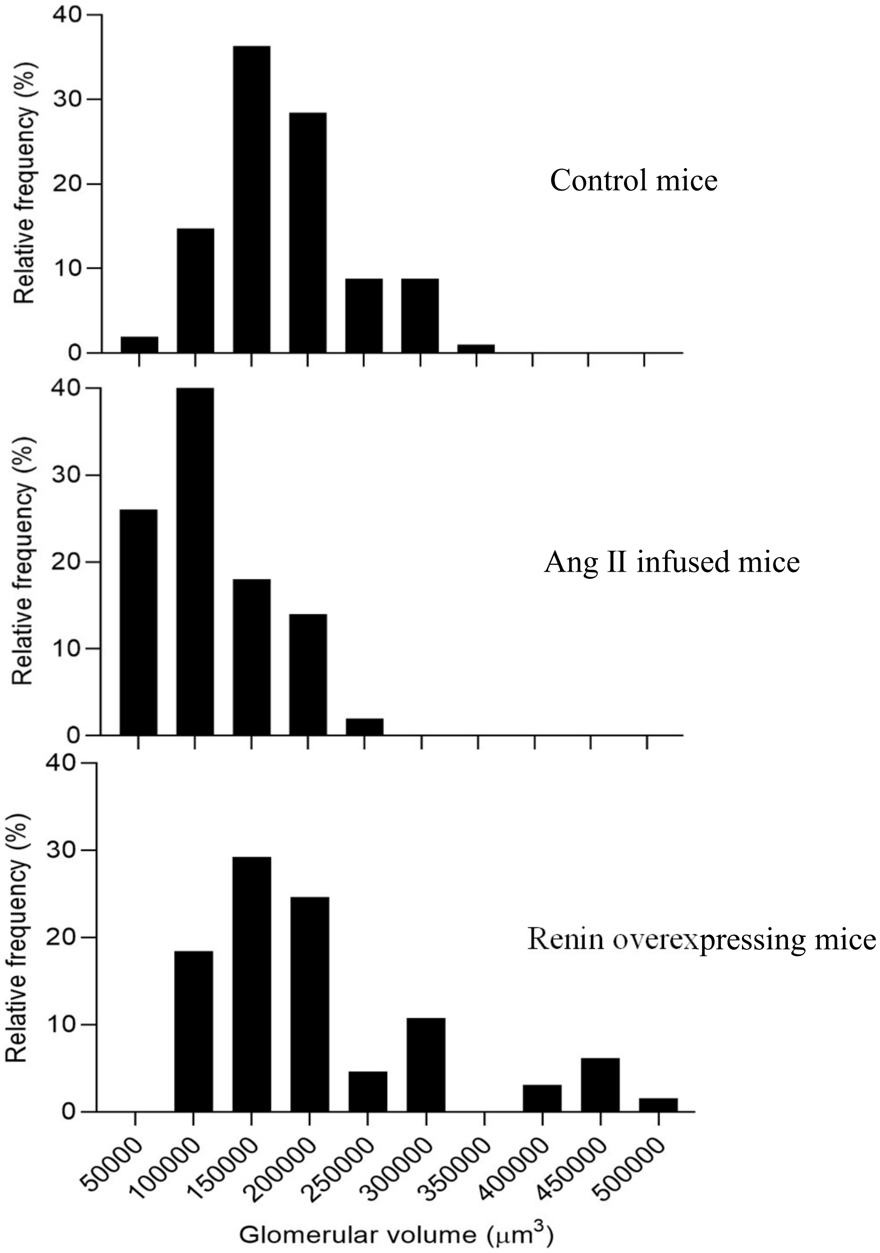

S1 Figure. The glomerular volume distribution in mouse models. Control mice demonstrated the normal distribution curve ($P>0.001$). In the angiotensin II infused mice the decreased mean glomerular volume explains the left shifted curve ($P<0.001$). In the renin overexpressing mice the right asymmetry with outliers represents a pool of hypertrophic glomeruli ($P<0.0001$).
