# Supplementary material for: Comparative analysis of hypertensive nephrosclerosis in animal models of hypertension and its relevance to human pathology. Glomerulopathy
Source: PLoS One. 2022 Feb 17;17(2):e0264136. doi: 10.1371/journal.pone.0264136 (PMC8853553; doi:10.1371/journal.pone.0264136)
Supplement: S2 Fig — The normal distribution curve characterises control rats (P>0.0001). The asymmetric right shifted curve and outliers in spontaneously hypertensive rats is the results of the increased glomerular volume (P<0.0001). The augmented glomerular volume in the nonclipped kidneys results in a normal distribution (P>0.001). The distribution curve in the clipped kidney is sharply left shifted because of numerous ischemic collapsed glomeruli (P<0.0001). (PDF) [file pone.0264136.s002.pdf]

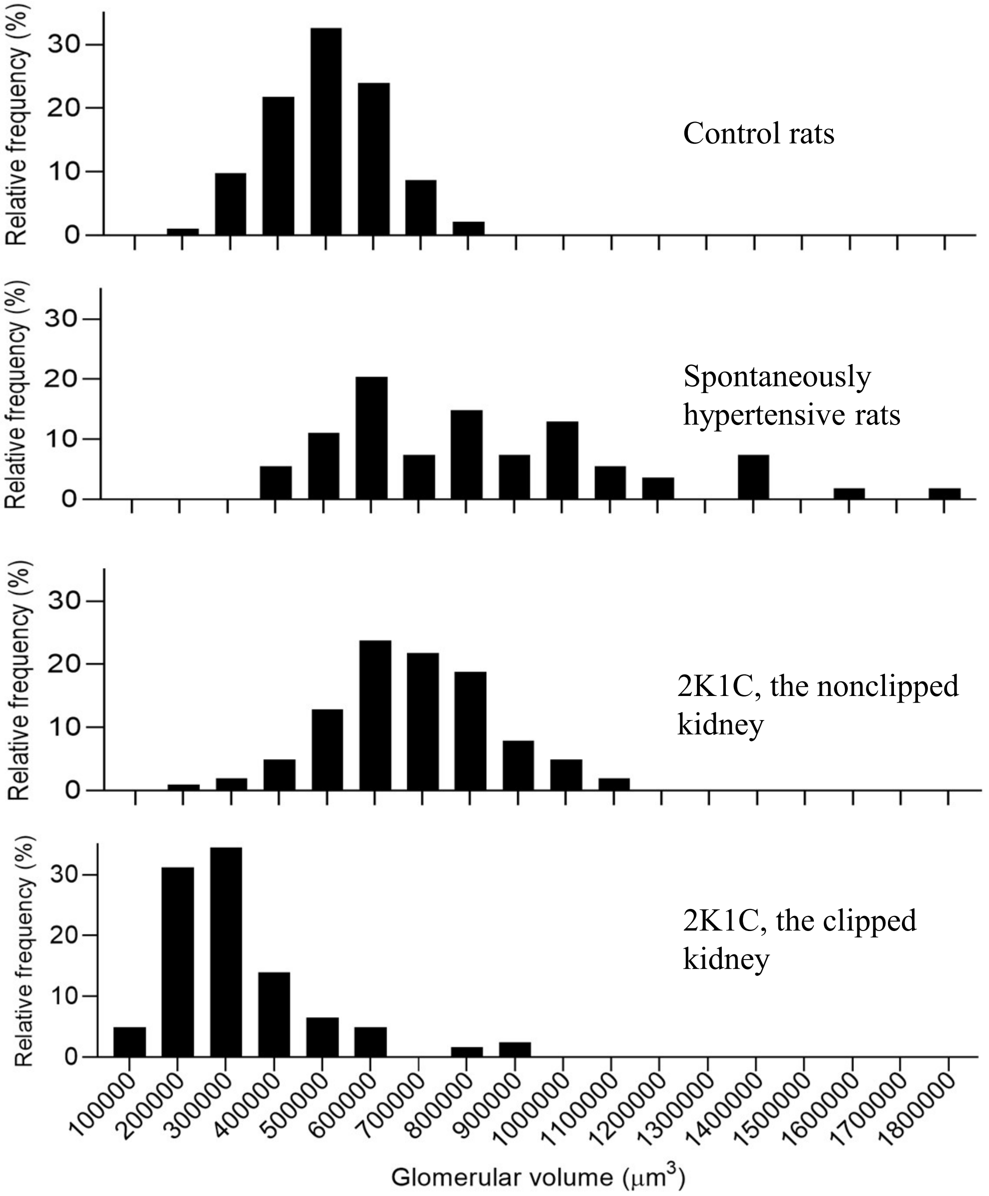

S2 Figure. The glomerular volume distribution in rat models. The normal distribution curve characterises control rats (P>0.0001). The asymmetric right shifted curve and outliers in spontaneously hypertensive rats is the results of the increased glomerular volume (P<0.0001). The augmented glomerular volume in the nonclipped kidneys results in a normal distribution (P>0.001). The distribution curve in the clipped kidney is sharply left shifted because of numerous ischemic collapsed glomeruli (P<0.0001).
