# Supplementary material for: Comparative analysis of hypertensive nephrosclerosis in animal models of hypertension and its relevance to human pathology. Glomerulopathy
Source: PLoS One. 2022 Feb 17;17(2):e0264136. doi: 10.1371/journal.pone.0264136 (PMC8853553; doi:10.1371/journal.pone.0264136)
Supplement: S3 Fig — Extent of involvement of the cortical area varies among animals. They could be minimal (3–5% of the renal cortex, upper image, animal #0524) or occupy the entire cortex (60–80% of the renal cortex, lower image, animal # 0341) Scale 500 μm, x25, hematoxylin-eosin. (PDF) [file pone.0264136.s003.pdf]

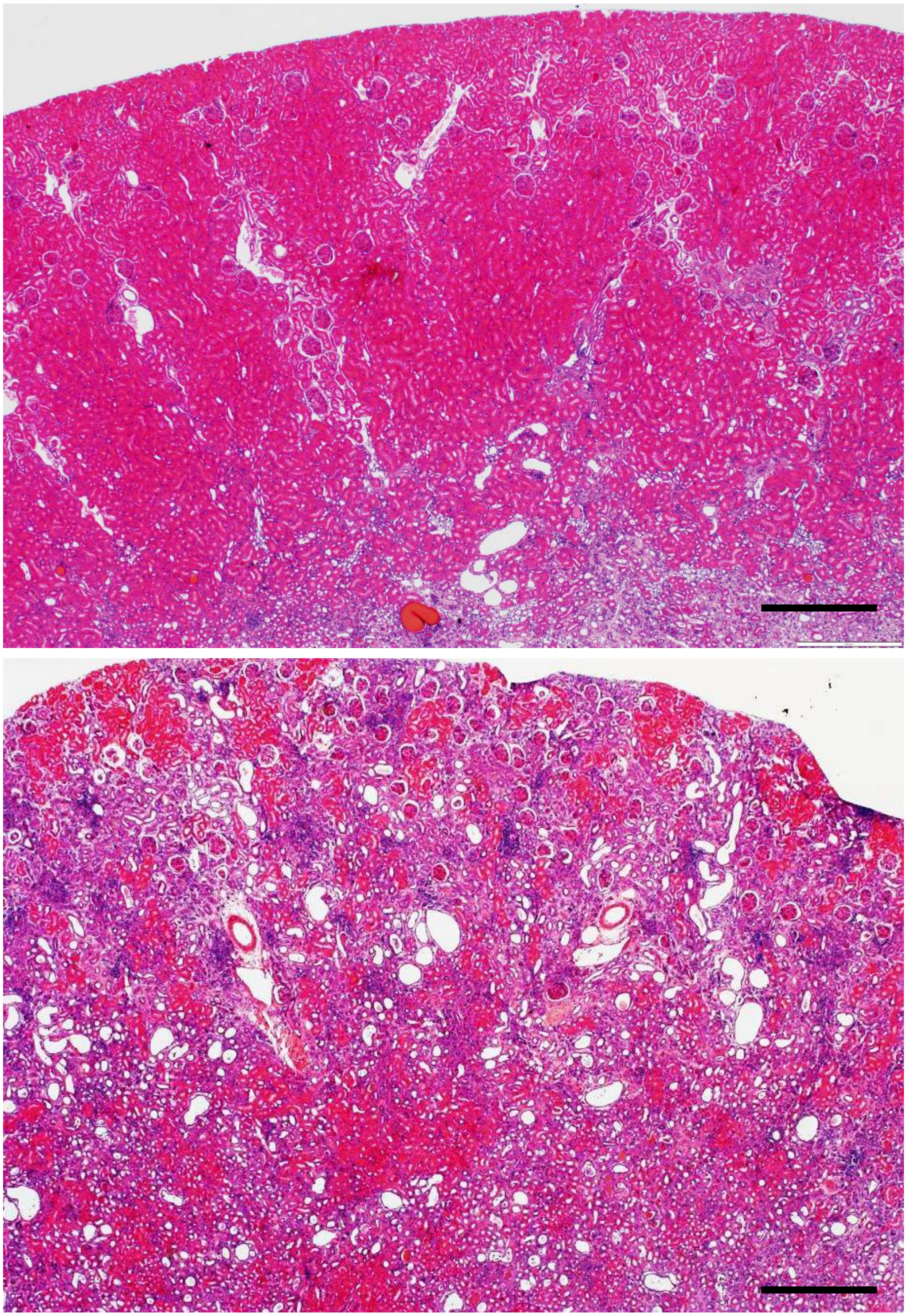

S3 Figure. Variable appearance of histopathological lesions in the renal cortex of the clipped kidney. Extent of involvement of the cortical area varies among animals. They could be minimal (3-5% of the renal cortex, upper image, animal #0524) or occupy the entire cortex (60-80% of the renal cortex, lower image, animal # 0341) Scale 500 µm, x25, hematoxylin-eosin.
