# Supplementary material for: Comparative analysis of hypertensive nephrosclerosis in animal models of hypertension and its relevance to human pathology. Glomerulopathy
Source: PLoS One. 2022 Feb 17;17(2):e0264136. doi: 10.1371/journal.pone.0264136 (PMC8853553; doi:10.1371/journal.pone.0264136)
Supplement: S1 Table — (PDF) [file pone.0264136.s004.pdf]

S1 Table. Diversity of the glomerulosclerosis score in animal studies

Author/models	Scoring method	Pathological variables	Formula	Range
Raij et al. [1]<br>Dahl rats	1 (<25%)<br>2 (<50%)<br>3 (<75%)<br>4 (100%)	Cells rarefaction<br>Capillary collapses<br>GBM folding<br>Mesangial expansion	$GLS = 100\left(\frac{1n_1}{N}+\frac{2n_2}{N}+\frac{3n_3}{N}+\frac{4n_4}{N}\right)$	0 - 400
Olson et al. [2]<br>Aortic ligature,<br>DOCA-salt, SHR	1 (<25%)<br>2 (<75%)<br>3 (>75%)	Mesangial expansion<br>Mesangial sclerosis,<br>Mesangial hyalinosis	$GLS = \frac{1n_1+2n_2+3n_3}{N}$	0 - 3
Nahas et al. [3]<br>Subtotal nephrectomy	1 (light)<br>2 (<50%)<br>3 (>50%)<br>4 (total)	Mesangial expansion<br>GBM thickening<br>Hyalinosis<br>Sclerosis	$GLS = \frac{1n_1+2n_2+3n_3+4n_4}{N}$	0 - 4
Veniant et al. [4]<br>2K1C	1 (<25%)<br>2 (25-50%)<br>3 (50-75%)<br>4 (>75%)	Mesangial sclerosis<br>Mesangial hyalinosis	$GLS = 1n_1+2n_2+3n_3+4n_4$	0 - 400
Eirin et al. [5]<br>2K1C	Number of sclerotic glomeruli	Sclerotic glomeruli	$GLS = \frac{n}{N}\,100$	0 - 100
Pillebout et al. [6]<br>Subtotal nephrectomy	1 (light)<br>2 (severe)	Mesangial cell proliferation<br>Mesangial hyalinosis<br>Mesangial sclerosis	$GLS = \frac{1n_1+2n_2}{N}$	0 - 2
Gandhi et al. [7]<br>Subtotal nephrectomy	< 50% or >50% of glomerular area	Capillary collapses<br>Hyalinosis<br>Adhesions	Glomeruli with < 50% sclerosis, %<br>Glomeruli with > 50% sclerosis, %<br>Glomeruli with global sclerosis, %	0 - 100<br>0 - 100<br>0 - 100
Hewitson et al. [8]<br>Aortic ligature, DOCA	Number of glomeruli	Fibrinoid<br>Crescents<br>Epithelial cell droplets	Percentage of glomeruli	0-100
Griffin et al. [9]<br>SHR	Number of glomeruli	Ischemic glomeruli<br>Acute hypertensive injury<br>Segmental sclerosis	Percentage of glomeruli	0-100
Konopka et al. [10]<br>2K1C	Number of glomeruli	Glomerular collapse<br>Glomerular hypertrophy<br>Glomerular sclerosis	Percentage of glomeruli	0-100
Esteban et al. [11]<br>SHR, nephrectomy	1 (<25%)<br>2 (25-50%)<br>3 (50-75%)<br>4 (>75%)	Mesangial expansion<br>Mesangial cell proliferation	Area percentage of the sample	0-100

SHR – spontaneously hypertensive rats; DOCA – deoxycorticosterone acetate; 2K1C – two-kidney one-clip; N - total number of counted glomeruli; n - number of glomeruli with the corresponding score of lesions; GLS – glomerular lesion score; GBM – glomerular basement membrane.

S1 Table. References

1. Raij L, Azar S, Keane W. Mesangial immune injury, hypertension, and progressive glomerular damage in Dahl rats. Kidney Int 1984; 26: 137–43.

2. Olson JL, Wilson SK, Heptinstall RH. Relation of glomerular injury to preglomerular resistance in experimental hypertension. Kidney Int 1986; 29: 849–857.

3. Nahas AM, Bassett AH, Cope GH, et al. Role of growth hormone in the development of experimental renal scarring. Kidney Int 1991; 40: 29–34.

4. Véniant M, Heudes D, Clozel JP, et al. Calcium blockade versus ACE inhibition in clipped and unclipped kidneys of 2K-1C rats. Kidney Int 1994; 46: 421–429.

5. Eirin A, Li Z, Zhang X, et al. A mitochondrial permeability transition pore inhibitor improves renal outcomes after revascularization in experimental atherosclerotic renal artery stenosis. Hypertens (Dallas, Tex 1979) 2012; 60: 1242–9.

6. Pillebout E, Weitzman JB, Burtin M, et al. JunD protects against chronic kidney disease by regulating paracrine mitogens. J Clin Invest 2003; 112: 843–852.

7. Gandhi M, Olson JL, Meyer TW. Contribution of tubular injury to loss of remnant kidney function. Kidney Int 1998; 54: 1157–1165.

8. Hewitson T, Kamitsuji H, Whitworth JA, et al. Comparison of the vascular and glomerular changes in aortic-ligature and DOCA-salt hypertension. Clin Exp Pharmacol Physiol 1989; 16: 641–645.

9. Griffin KA, Churchill PC, Picken M, et al. Differential salt-sensitivity in the pathogenesis of renal damage in SHR and stroke prone SHR. Am J Hypertens 2001; 14: 311–320.

10. Konopka CL, Jurach A, Wender OCB. Experimental model for the study of chronic renal ischemia in rats. Morphologic, histological and ultra-structural analysis. Acta Cir Bras 2007; 22: 12–21.

11. Esteban V, Galelego-Delgado J, Lazaro A, Osende J, Mezzano S, Egido J R-OM. Long-term treatment with an ACE inhibitor or an AT1 antagonist avoids hypertension-induced inflammation in the kidney. J Nephrol Nov-Dec 2006;19(6)725-31 2006; 19: 725–31.
