# Supplementary material for: Comparative analysis of hypertensive nephrosclerosis in animal models of hypertension and its relevance to human pathology. Glomerulopathy
Source: PLoS One. 2022 Feb 17;17(2):e0264136. doi: 10.1371/journal.pone.0264136 (PMC8853553; doi:10.1371/journal.pone.0264136)
Supplement: S2 Table — (PDF) [file pone.0264136.s005.pdf]

S2 Table. Semi-quantitative values or definitions of severity/distribution for pathological variables in the Oxford classification of IgA nephropathy*

Whole glome-rulus	Mesangial cell hypercellularity	Endo-capillary hyper-cellularity	Extra-capillary lesions - cellular	Extra-capillary lesions - fibrocellular	Extra-capillary lesions - fibrous
Normal glomerulus	No hypercellularity (0)	Segmental	Tiny focus (<10%)	Tiny focus (<10%)	Crescent (10-25%)
Segmental sclerosis	Mild (1) 4-5 cells	Global	Crescent (10-25%)	Crescent (10-25%)	Crescent (26-50%)
Adhesion	Moderate (2) 6-7 cells	GBM duplication	Crescent (26-50%)	Crescent (26-50%)	Crescent (>50%)
Ischemia/ /Collapse	Severe (3) (≥8 cells)	Necrosis	Crescent (>50%)	Crescent (>50%)	

* Roberts ISD, Cook HT, Troyanov S, et al. The Oxford classification of IgA nephropathy: Pathology definitions, correlations, and reproducibility. Kidney Int 2009; 76: 546–556.
