# Supplementary material for: Comparative analysis of hypertensive nephrosclerosis in animal models of hypertension and its relevance to human pathology. Glomerulopathy
Source: PLoS One. 2022 Feb 17;17(2):e0264136. doi: 10.1371/journal.pone.0264136 (PMC8853553; doi:10.1371/journal.pone.0264136)
Supplement: S3 Table — (PDF) [file pone.0264136.s006.pdf]

S3 Table. Semi-quantitative values or definitions of severity/distribution for pathological variables in the classification of diabetic nephropathy*

Class I	Class IIa	Class IIb	Class III	Class IV
**Mild or non-specific LM changes and EM-proven GBM thickening**	**Mild mesangial expansion**	**Severe mesangial expansion**	**Nodular sclerosis (Kimmelstiel–Wilson lesion)**	**Advanced diabetic glomerulo-sclerosis**
Biopsy does not meet any of the criteria mentio-ned below for class II, III, or IV; GBM > 395 nm in female and > 430 nm in male individuals	Biopsy does not meet criteria for class III or IV; mild mesangial expansion in >25% of the observed mesangium	Biopsy does not meet criteria for class III or IV; severe mesangial expansion in >25% of the observed mesangium	Biopsy does not meet criteria for class IV; at least one convincing Kimmelstiel–Wilson lesion	Global glomerular sclerosis in >50% of glomeruli; lesions from classes I through III

*Tervaert TWC, Mooyaart AL, Amann K, et al. Pathologic classification of diabetic nephropathy. J Am Soc Nephrol 2010; 21: 556–563. LM – light microscopy; EM – electron microscopy; GBM – glomerular basement membrane
