# Supplementary material for: Comparative analysis of hypertensive nephrosclerosis in animal models of hypertension and its relevance to human pathology. Glomerulopathy
Source: PLoS One. 2022 Feb 17;17(2):e0264136. doi: 10.1371/journal.pone.0264136 (PMC8853553; doi:10.1371/journal.pone.0264136)
Supplement: S4 Table — (PDF) [file pone.0264136.s007.pdf]

S4 Table. Semi-quantitative values or definitions of severity/distribution in the classification of focal segmental glomerulosclerosis *

FSGS (NOS)	Perihilar variant	Cellular variant	Tip variant	Collapsing variant
At least 1 glomerulus with segmental increase in matrix obliterating the capillary lumina. There may be segmental glomerular capillary wall collapse without overlying podocyte hyperplasia	At least 1 glomerulus with perihilar hyalinosis, with or without sclerosis. >50% of glomeruli with segmental lesions must have perihilar sclerosis and/or hyalinosis	At least 1 glomerulus with segmental endocapillary hypercellularity occluding lumina, with or without foam cells and karyorrhexis	At least 1segmental lesion involving the tip domain (outer 25% of tuft next to origin of proximal tubule). The tubular pole must be identified in the defining lesion. The lesion must have either an adhesion or confluence of podocytes with parietal or tubular cells at the tubular lumen or neck. The tip lesion may be cellular or sclerosing	At least 1 glomerulus with segmental or global collapse and overlying podocyte hypertrophy and hyperplasia

*D'Agati VD, Fogo AB, Bruijn JA, et al. Pathologic classification of focal segmental glomerulosclerosis: a working proposal. Am J Kidney Dis 2004; 43: 368–382. FSGS – focal segmental glomerulosclerosis; NOS – not otherwise specified.
